# Reprogramming of gene expression during compression wood formation in pine: Coordinated modulation of S-adenosylmethionine, lignin and lignan related genes

**DOI:** 10.1186/1471-2229-12-100

**Published:** 2012-06-29

**Authors:** David P Villalobos, El-Sayed S Said, Rafael A Cañas, Sonia H E Van Kerckhoven, Rocío Bautista, Manuel Gonzalo Claros, Francisco M Cánovas, Francisco R Cantón

**Affiliations:** 1 Departamento de Biología Molecular y Bioquímica, Facultad de Ciencias, Universidad de Málaga, Campus Universitario de Teatinos, 29071, Málaga, Spain; 2 Department of Plant Molecular Biology, University of Lausanne, CH-1015, Lausanne, Switzerland; 3 Division of Glycoscience, School of Biotechnology, Royal Institute of Technology, AlbaNova University Centre, SE-10691, Stockholm, Sweden; 4 Departamento de Fisiología Vegetal, Centro Hispano-Luso de Investigaciones Agrarias, Facultad de Biología, Universidad de Salamanca, C/Río Duero 12, 37185, Salamanca, Spain

## Abstract

**Background:**

Transcript profiling of differentiating secondary xylem has allowed us to draw a general picture of the genes involved in wood formation. However, our knowledge is still limited about the regulatory mechanisms that coordinate and modulate the different pathways providing substrates during xylogenesis. The development of compression wood in conifers constitutes an exceptional model for these studies. Although differential expression of a few genes in differentiating compression wood compared to normal or opposite wood has been reported, the broad range of features that distinguish this reaction wood suggest that the expression of a larger set of genes would be modified.

**Results:**

By combining the construction of different cDNA libraries with microarray analyses we have identified a total of 496 genes in maritime pine (*Pinus pinaste*r, Ait.) that change in expression during differentiation of compression wood (331 up-regulated and 165 down-regulated compared to opposite wood). Samples from different provenances collected in different years and geographic locations were integrated into the analyses to mitigate the effects of multiple sources of variability. This strategy allowed us to define a group of genes that are consistently associated with compression wood formation. Correlating with the deposition of a thicker secondary cell wall that characterizes compression wood development, the expression of a number of genes involved in synthesis of cellulose, hemicellulose, lignin and lignans was up-regulated. Further analysis of a set of these genes involved in S-adenosylmethionine metabolism, ammonium recycling, and lignin and lignans biosynthesis showed changes in expression levels in parallel to the levels of lignin accumulation in cells undergoing xylogenesis *in vivo* and *in vitro*.

**Conclusions:**

The comparative transcriptomic analysis reported here have revealed a broad spectrum of coordinated transcriptional modulation of genes involved in biosynthesis of different cell wall polymers associated with within-tree variations in pine wood structure and composition. In particular, we demonstrate the coordinated modulation at transcriptional level of a gene set involved in S-adenosylmethionine synthesis and ammonium assimilation with increased demand for coniferyl alcohol for lignin and lignan synthesis, enabling a better understanding of the metabolic requirements in cells undergoing lignification.

## Background

Large amounts of wood can be formed throughout the life of a tree through a complex process of cell differentiation called xylogenesis. In this process, cambium-derived cells undergo cell division followed by thickening of the secondary cell wall by modification of the synthesis and deposition of cellulose, hemicelluloses, cell wall proteins and lignin, and finally programmed cell death to develop tracheary elements [1]. Genes involved in these cellular processes are under strict transcriptional regulation during different stages of differentiation [2]. Nevertheless, inputs from external cues are also integrated in this developmental program to adapt secondary xylem properties to growth requirements in a continuously changing environment [3]. As a result of this interaction, natural variations are found in wood properties not only among different species and genotypes, but also within the same tree [4].

Transcriptome analysis of differentiating secondary xylem has allowed us to draw a general picture of the genes, metabolic pathways and potential regulators involved in wood formation [5-11]. However, our knowledge is limited as to how the transcriptomes, proteomes and metabolomes involved in wood development are modulated by developmental and environmental signals to cause within-tree variation in wood properties. Considerable effort has been focused on studying the main pathways that lead to monolignol biosynthesis [12] and carbohydrate partitioning to cellulose [13], as well as understanding how changes in gene expression in those pathways may affect cellular wall characteristics and, consequently, wood quality [14]. However, less attention has been paid to the pathways that provide S-adenosylmethionine (SAM) for methylation reactions during biosynthesis of coniferyl and sinapyl alcohols, even though transcripts and proteins for enzymes of these pathways are abundant in the developing xylem [15,16]. Consumption of methyl units during lignification also implies the existence of an important carbon sink, and modifications in the availability of SAM may affect wood quality through alterations in lignin content and composition [16-18]. Furthermore, it has been proposed that glycine decarboxylase activity associated with SAM metabolism could be an important source of released ammonium, which must be efficiently re-assimilated to prevent significant imbalances in the strict nitrogen economy of the plant [19].

Molecular determinants of intra-genotype variation can be identified by comparing transcriptomes from cambial derivatives undergoing differentiation in the same tree that produce wood that differs in composition and structure [4]. However, the plasticity of the molecular machinery involved in wood formation [3] may result in some difficulties in terms of defining candidate genes when expression patterns are compared over different annual growth periods. Therefore, the definition of a consistent set of candidate genes for wood property variations requires analysis that mitigates sources of variation from genotypes and local environmental changes.

Reaction wood illustrates how the integration of environmental signals into the secondary xylem developmental program leads to intra-genotype variation in wood. When the stem of a woody plant grows in a non-vertical orientation it forms reaction wood, a specialized secondary xylem that helps the stem maintain a certain orientation and re-orients growth [20]. In gymnosperms, reaction wood is called compression wood and it develops on the underside of branches and inclined stems [21]. Cell walls in compression wood tracheids are thicker than in normal wood and lack the innermost S3 layer. It also contains more lignin, less cellulose and altered levels of hemicelluloses than normal wood. Moreover, in compression wood cellulose microfibrils are deposited with increased angle relative to the cell axis [22,23], and the lignin is enriched in p-hydroxyphenylpropane units [24,25]. In contrast, opposite wood develops on the opposite side of the inclined stem and it is more similar to normal wood [25]. Differential expression of a few genes during compression wood differentiation compared to normal or opposite wood has been reported [16,26-28]. However, the clear anatomical, structural and compositional differences that characterize compression wood suggest that the expression of more genes would be modified during its differentiation [25,29,30].

We are interested in how biosynthetic pathways that provide substrates for xylogenesis are coordinated and regulated according to the different demands during development of wood with distinct composition. In particular, comparison of samples from differentiating compression wood and opposite wood from the same tree allows us to analyze the transcriptome changes accompanying to different levels of lignin deposition during xylogenesis. In this work, we present the results of a comparative transcriptomic analysis that comprehensively uses a range of cDNA libraries and microarray analyses, combining samples from different *Pinus pinaste*r provenances collected in different years and geographic locations, to identify changes in the transcriptome associated with compression wood development.

## Results

### Identification of genes differentially expressed during compression and opposite wood formation in *Pinus pinaster*

**Figure 1:**
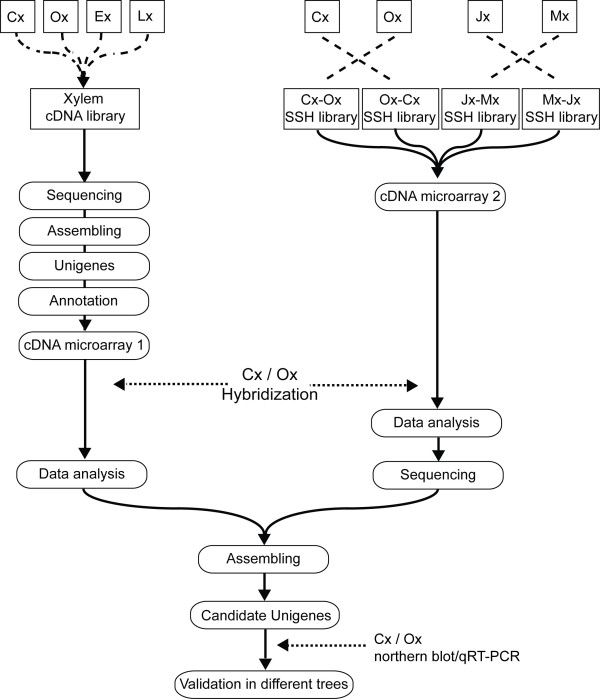
**Schematic overview of the experimental procedure used in this work to identify genes differentially expressed between compression and opposite developing xylem from maritime pine.** Samples of differentiating secondary xylem forming compression (Cx), opposite (Ox), early (Ex), late (Lx), juvenile (Jx) and mature (Mx) wood were used for cDNA libraries construction.

To identify genes that are differentially expressed between compression (Cx) and opposite (Ox) developing xylem we followed the strategy depicted in Figure 1. Samples from different provenances collected in different years at two latitudes were integrated into the analyses to mitigate the effects of multiple sources of variability. Two microarrays were manufactured with cDNA libraries constructed with RNA isolated from adult maritime pine trees of Corsican and Aquitaine provenances grown in Aquitaine, France. The first microarray (Figure 1, cDNA microarray 1) was constructed using a set of 2800 cDNAs from samples of Cx, Ox, early (Ex) and late (Lx) developing xylem collected in 1998, 1999 and 2000. The second microarray (Figure 1, cDNA microarray 2) was constructed with 4041 cDNA clones from four subtractive libraries obtained by reciprocally subtracting cDNA populations obtained from Cx and Ox samples that were collected in 1998 and from juvenile (Jx) and mature (Mx) developing xylem collected in 2001. Both microarrays were hybridized with labeled aRNA targets synthesized from Cx and Ox collected from four maritime pine trees of the Sierra Bermeja provenance, Spain [31], sampled in 2005. To validate the expression patterns of selected genes, northern blot and qRT-PCR analyses were carried out with samples of adult trees from Sierra Bermeja provenance collected in 2005 and 2008, respectively.

**Figure 2:**
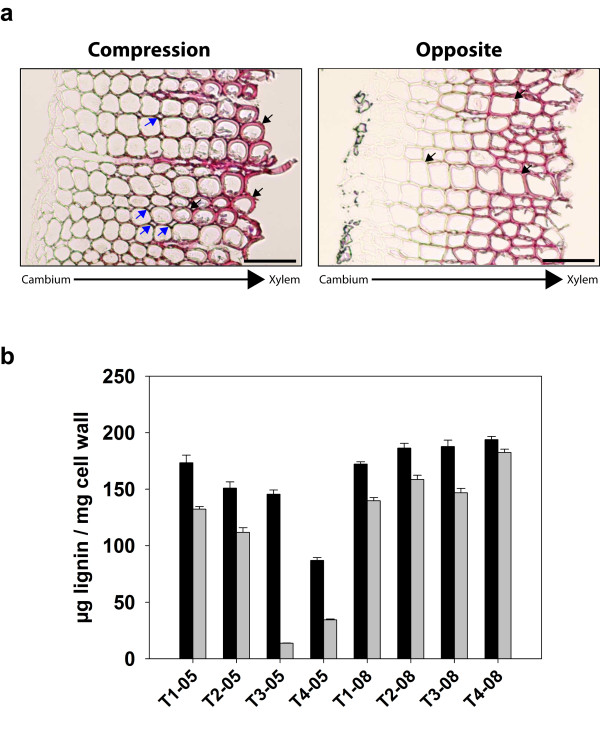
**Cross-sectional images and lignin content analysis of cambial scraping samples used in gene expression analyses.** (a) Representative microscopic images of thin transversal cross-sections of cambial scrapings stained with phloroglucinol. The orientation of the differentiating tracheids in the scraping relative to the cambium and developed xylem is indicated at the bottom of the pictures. Blue arrows mark typical intercellular spaces in compression wood, and black arrows mark cells with characteristic rounded shape in compression wood or straight sides in opposite wood. Scale bar at the bottom left represents 0.1 mm. (b) Lignin content of cambial scrapings from compression (black bars) and opposite (grey bars) sides of the eight different maritime pine trees analyzed, sampled in May 2005 (T1-05, T2-05, T3-05, T4-05) and May 2008 (T1-08, T2-08, T3-08, T4-08) after two months of artificial mechanical bending. Lines on the top of every bar show standard error of the mean for four independent replicates. Statistical significance of differences in lignin content between cambial scrapings from compression and opposite sides from the same tree was determined with a paired Student´s *t*-test (p < 0.05). All differences were statistically significant except for tree T4-08.

Microscopic examination and lignin quantification were performed for phenotypic validation of the samples (Figure 2). Consistent with the typical morphology of compression wood tracheids [25], transversal sections of developing tracheids showed a rounded shape and many intercellular spaces, whereas Ox developing tracheids had straight sides and few intercellular spaces (Figure 2a). Lignin content was determined in samples from the four trees used in microarray analyses and the four trees used in qRT-PCR (Figure 2b). Cx samples contained significantly more lignin than Ox samples. However, variability in lignin content between both types of developing xylem was evident among trees. In particular, the difference in lignin content between Cx and Ox samples from tree T4-08 was lower compared to the other trees and not statistically significant according to a *t*-test.

**Figure 3:**
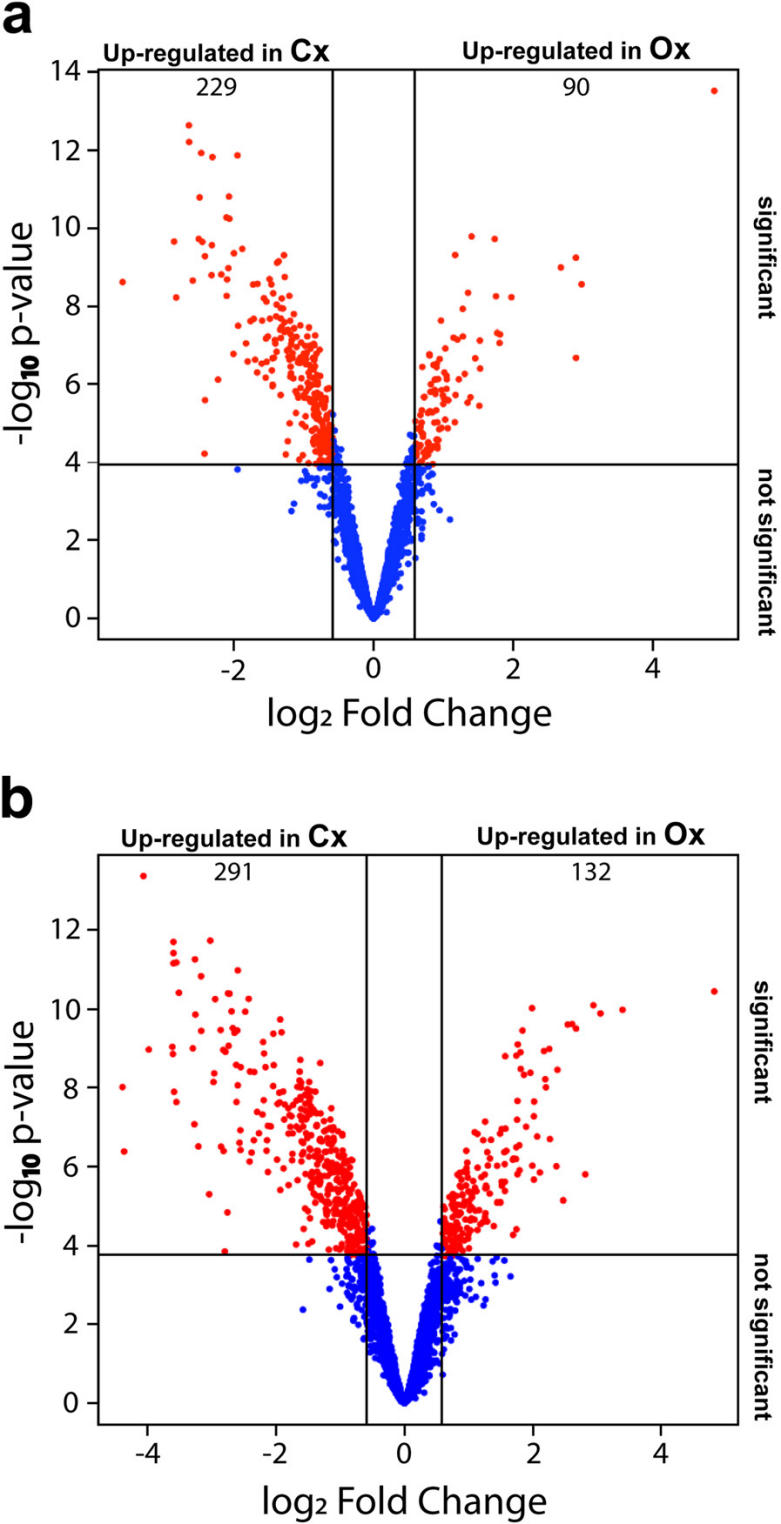
**Volcano plots of microarray analyses to identify genes differentially expressed during compression and opposite wood formation.** The common logarithm of the *p*-value was represented as a function of the binary logarithm of the background-corrected and normalized opposite:compression fluorescence ratio (log_2_ Fold Change) for each spot. Vertical bars delimit the spots showing up-regulation in developing compression xylem by at least 1.5-fold compared to developing opposite xylem (Up-regulated in Cx) or spots showing up-regulation in developing opposite xylem by at least 1.5-fold compared to developing compression xylem (Up-regulated in Ox). The horizontal line delimits the spots showing significant up-regulation under the criteria of an adjusted *p*-value ≤ 0.001. Therefore, the upper left and right sectors delimited by the horizontal and vertical lines include the spots (in red) containing probes for genes significantly up-regulated in developing compression or opposite xylem respectively. The number of spots corresponding with genes significantly up-regulated in Cx or Ox are shown in the top side of the sector. (a) Results from the analysis of microarray 1 constructed with cDNA clones from the composite library. (b) Results from the analysis of microarray 2 constructed with cDNA clones from subtractive libraries.

Results of hybridization analyses of both microarrays are summarized as volcano plots in Figure 3. After eliminating spots flagged as bad, not found or absent and those with a signal intensity that did not surpass twofold their background signal, only 2598 and 3148 spots were kept for further analysis in microarray 1 and 2 respectively. Relative expression values (log_2_ Fold Change), *p*-values and adjusted *p*-values for all the spots represented in Figure 3 are available in Additional files 1 and 2. A clear bias is observed in both cases, with a higher number of spots showing up-regulation in Cx compared to Ox. The analysis of microarray 1 shows 229 spots containing probes for genes that were significantly up-regulated (*p* ≤ 0.001) by at least 1.5-fold during compression wood formation, and only 90 spots showing up-regulation during opposite wood formation (Figure 3a). Since these cDNAs were previously sequenced to construct an EST database of *Pinus pinaster* developing xylem the sequences were already available [32]. The analysis of microarray 2 identified 291 spots showing up-regulation in Cx and 132 spots showing up-regulation in Ox (Figure 3b). Only these 423 cDNA clones were sequenced from the probe set of microarray 2.

Sequences with an average Phred score >20 per nucleotide in a sliding window of 15 nucleotides and a minimal length of 100 nucleotides were kept for further analysis (average length of 437 nucleotides and a standard deviation of ±171). Those with lower quality scores or shorter than 100 nucleotides were re-sequenced. The complete set of sequences corresponding to differentially expressed genes from microarray 1 and 2 were used to define a set of unigenes, and a total number of 355 unigenes up-regulated in Cx and 176 unigenes up-regulated in Ox were obtained. Moderate values of sequence redundancy were obtained (31.6% for Cx and 21.2% for Ox).

**Figure 4:**
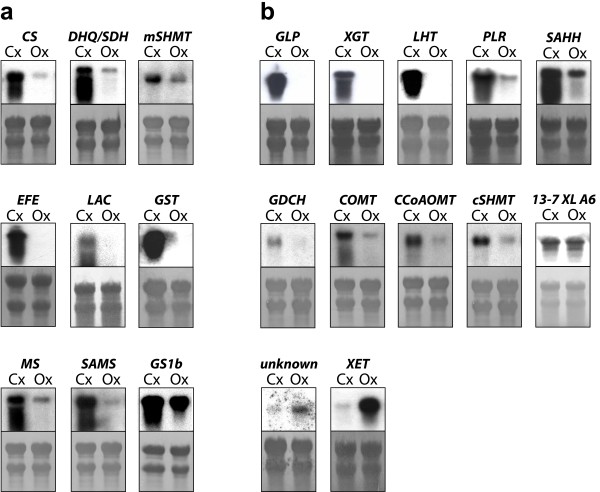
**Validation of the expression profiles of selected genes in compression and opposite developing xylem.** Equal amounts of total RNA isolated from compression (Cx) and opposite (Ox) differentiating xylem from adult maritime pine trees were size fractioned by electrophoresis and transferred to nylon membranes. PCR-amplified cDNA from 21 genes identified as differentially expressed in analyses of cDNA microarray 1 (a) or cDNA microarray 2 (b) were labeled with ^32^P and used as probes. The panel 13-7 XL A6 shows the result of hybridization with cDNA from a gene classified as non-differentially expressed by the microarray analyses. Panels below every northern blot show methylene blue staining, as a loading control. Functional annotation and accession number of the corresponding ESTs: *CS* (chorismate synthase, BX250851), *DHQ/SDH* (3-dehydroquinate dehydratase/shikimate 5-dehydrogenase, BX251808), *mSHMT* (mitocondrial serine hydroxymethyltransferase, BX249820), *EFE* (ethylene-forming enzyme, BX251740), *LAC* (laccase, BX252150), *GST* (glutathione S-transferase, BX250376),* MS* (methionine synthase, BX255477), *SAMS* (S-adenosylmethionine synthetase, BX248790), *GS1b* (glutamine synthetase 1b, BX253698), *GLP* (germin-like protein, FN256510), *XGT* (xyloglucan galactosyltransferase, FN256497),* LHT* (lysine/histidine transporter, FN256535), PLR (pinoresinol-lariciresinol reductase, FN256693), *GDCH* (glycine decarboxylase complex H-protein, FN256878), *COMT* (caffeate O-methyltransferase, FN256925), *CCoAOMT* (caffeoyl-CoA-O-methyltransferase, FN256577), *cSHMT* (cytosolic serine hydroxymethyltransferase, FN256831), *SAHH* (S-adenosyl homocysteine hydrolase, FN256791),*unknown* (unknown-function gene, FN256975),* XET* (xyloglucan endotransglucosylase/hydrolase, FN256785), *13-7XLA6* (cDNA of a gene with the same level of expression in Cx and Ox according to microarray analyses).

To validate the transcriptomic analysis, a group of genes was selected and their cDNAs were used as probes for northern blot analysis of RNA extracted from Cx and Ox (Figure 4). The differential expression patterns in Cx and Ox observed in microarrays analyses were validated for all selected genes. In particular, northern blot analysis suggested that our strategy allowed us to identify some genes that may be specifically induced at high levels in Cx whereas are not expressed or expressed at very low levels in Ox (Figure 4, *EFE*, *GST*, *GLP*, *XGT*, *LHT*). As a control, the expression pattern was also confirmed for a gene that is expressed at similar levels in both types of differentiating xylem according to microarray analysis (Figure 4b, panel labeled *13-7XLA6*).

### Trancriptome changes in functional categories related to cell wall during compression wood formation in pine

The complete set of unigenes was functionally annotated using BlastX analysis [33] against GenBank and BlastN using the Pine Gene Index database (Additional file 3). Sequences that matched with the same entry in the database were assumed to represent the same gene. Therefore, the final numbers of unigenes were reduced to 331 for Cx and 165 for Ox. Most of these genes showed significant similarities to sequences in databases (293 in Cx and 145 in Ox), although some of them were similar to sequences with unknown function (49 in Cx and 45 in Ox). The number of unigenes with no significant similarity was low in both cases (38 in Cx and 20 in Ox).

**Figure 5:**
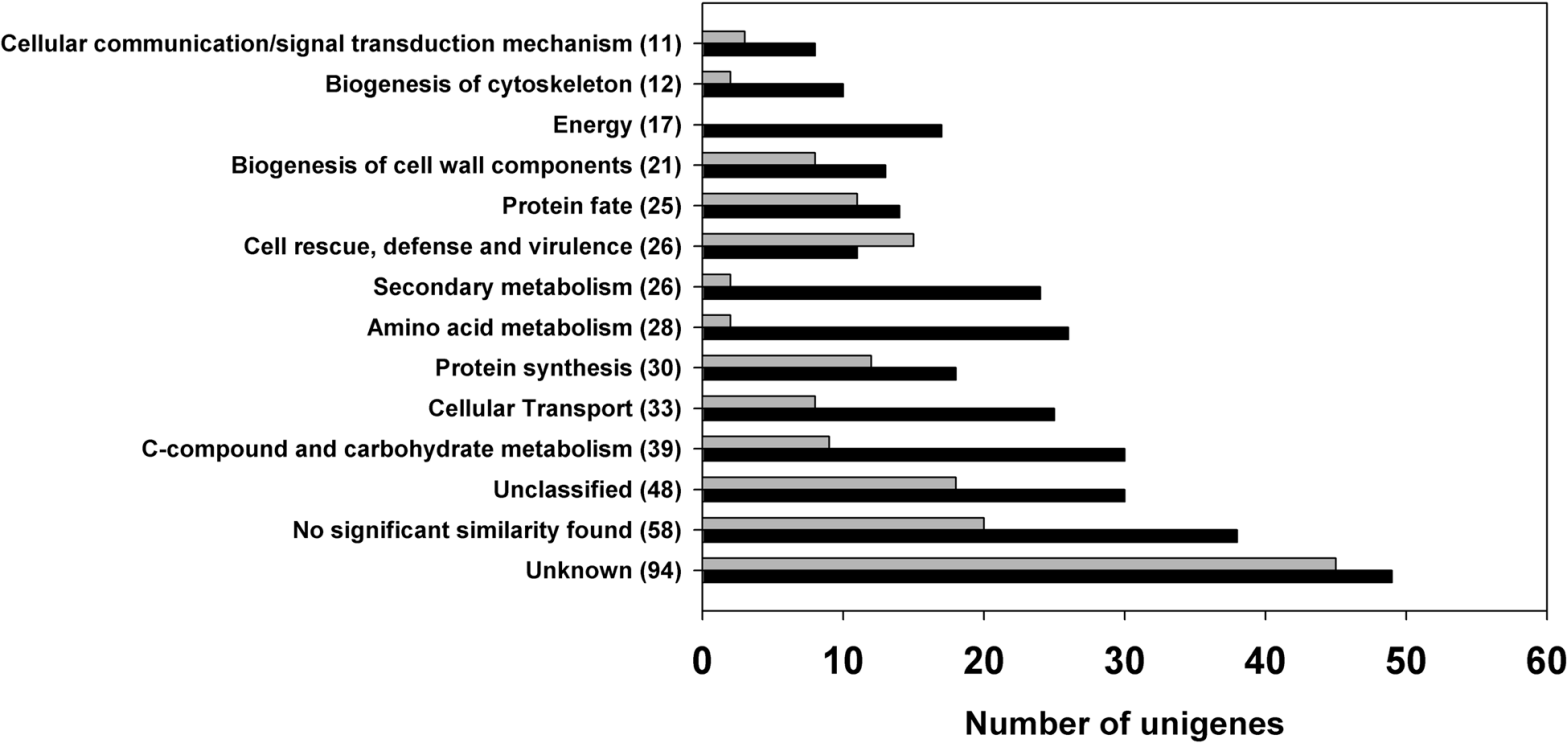
**The most represented functional categories among the genes differentially expressed in Cx and Ox.** The complete set of unigenes was grouped into functional categories using the MIPS database. Those categories with a total number of unigenes over 10 were considered as the most represented. The total number of unigenes is shown between brackets following the category name. The number of unigenes up-regulated in Cx is represented as black bars, and the number of unigenes up-regulated in Ox is represented as grey bars.

The genes with assigned function were grouped into functional categories using the *Arabidopsis thaliana* Munich Information Centre for Protein Sequences (MIPS) database, and suppression of redundancy in MIPS funcat assignations by decision according to their most probable role in xylem development (Additional file 3). In keeping with the greater number of genes identified as up-regulated in Cx, most of the functional categories included more genes in this tissue (Figure 5 and Additional file 3 for details). The most represented categories were “C-compound and carbohydrate metabolism” (30 in Cx/9 in Ox), “cellular transport” (25 in Cx/8 in Ox), “protein synthesis” (18 in Cx/12 in Ox), “amino acid metabolism” (26 in Cx/2 in Ox), “secondary metabolism” (24 in Cx/2 in Ox), “cell rescue, defense and virulence” (11 in Cx/15 in Ox) “protein fate” (14 in Cx/11 in Ox), “biogenesis of cell wall components” (13 in Cx/8 in Ox), “energy” (17 in Cx/0 in Ox), “biogenesis of cytoskeleton” (10 in Cx/2 in Ox), and “cellular communication/signal transduction mechanism” (8 in Cx/3 in Ox).

The functional categories with larger numbers of up-regulated genes in Cx compared to Ox are consistent with structural and chemical modifications of the cell wall, and as shown in Additional file 3 (“Up-regulated in Cx” tab) include genes encoding cellulose synthase subunits (contigs 19 and 7S, singletons BX249614 and FN256963), sucrose synthase (contigs 12 and 35S), cellulose synthase-like A (contig 19S), glycosyltransferases (contig 4P, singleton BX250177), xyloglucan galactosyltransferase (singleton FN256497), α- and β-tubulins (contigs 13, 7P, 31S and 51S, singletons BX255503, FN256632, FN256674, FN256784, BX255285, BX249611 and FN256695) and a putative microtubule-associated protein (singleton BX253390). In particular, the gene encoding xyloglucan galactosyltransferase (*XGT*) showed a high level of expression in Cx, whereas transcripts were undetectable in Ox (Figure 4b, *XGT*).

Transcripts for enzymes involved in lignin biosynthesis were also up-regulated in Cx (Additional file 3, “Up-regulated in Cx” tab), including those encoding enzymes in the monolignol biosynthetic pathway, such as phenylalanine ammonia lyase (PAL: contig 3S, singletons BX249569, BX253670 and FN257113), p-coumarate 3-hydroxylase (C3H: singleton BX248886), 4-coumarate:CoA ligase (4CL: singletons BX255483 and BX253310), cinnamyl alcohol dehydrogenase (CAD: contig 2P, singleton BX253572), hydroxycinnamoyl-CoA:shikimate hydroxycinnamoyl transferase (HCT, singleton FN256636), caffeoyl-CoA-O-methyltransferase (CCoAOMT, singleton FN256577) and caffeate O-methyltransferase (COMT, contig 19P).

Several genes up-regulated in Cx and classified in the functional category “amino acid metabolism” encode enzymes involved in the synthesis of precursors for monolignol biosynthesis, such as the enzymes of the main trunk of the shikimate pathway chorismate synthase (contig 5, singleton FN256628), 3-dehydroquinate dehydratase/shikimate 5-dehydrogenase (contig 22), shikimate kinase (singleton BX251461) and 5-enolpyruvylshikimate 3-phosphate synthase (singleton BX252482). In particular, the expression pattern was confirmed by northern blot for the genes encoding chorismate synthase, and 3-dehydroquinate dehydratase/shikimate 5-dehydrogenase (Figure 4a, *CS* and *DHQ/SDH*, respectively).

### Expression of genes encoding enzymes related to SAM biosynthesis in cells undergoing xylogenesis

Genes related to S-adenosylmethionine (SAM) metabolism were also widely represented among those up-regulated in Cx and classified in the functional category “amino acid metabolism” (Additional file 3, “Up-regulated in Cx” tab). SAM is a universal methyl donor for many different cellular metabolic reactions, including those catalyzed by CCoAOMT and COMT. Both enzymes are essential for the biosynthesis of coniferyl and sinapyl alcohols, precursors of lignin, lignans and other phenylpropanoids [34]. Therefore, the higher lignin content of compression wood may require a larger supply of SAM during development compared to opposite wood.

**Figure 6:**
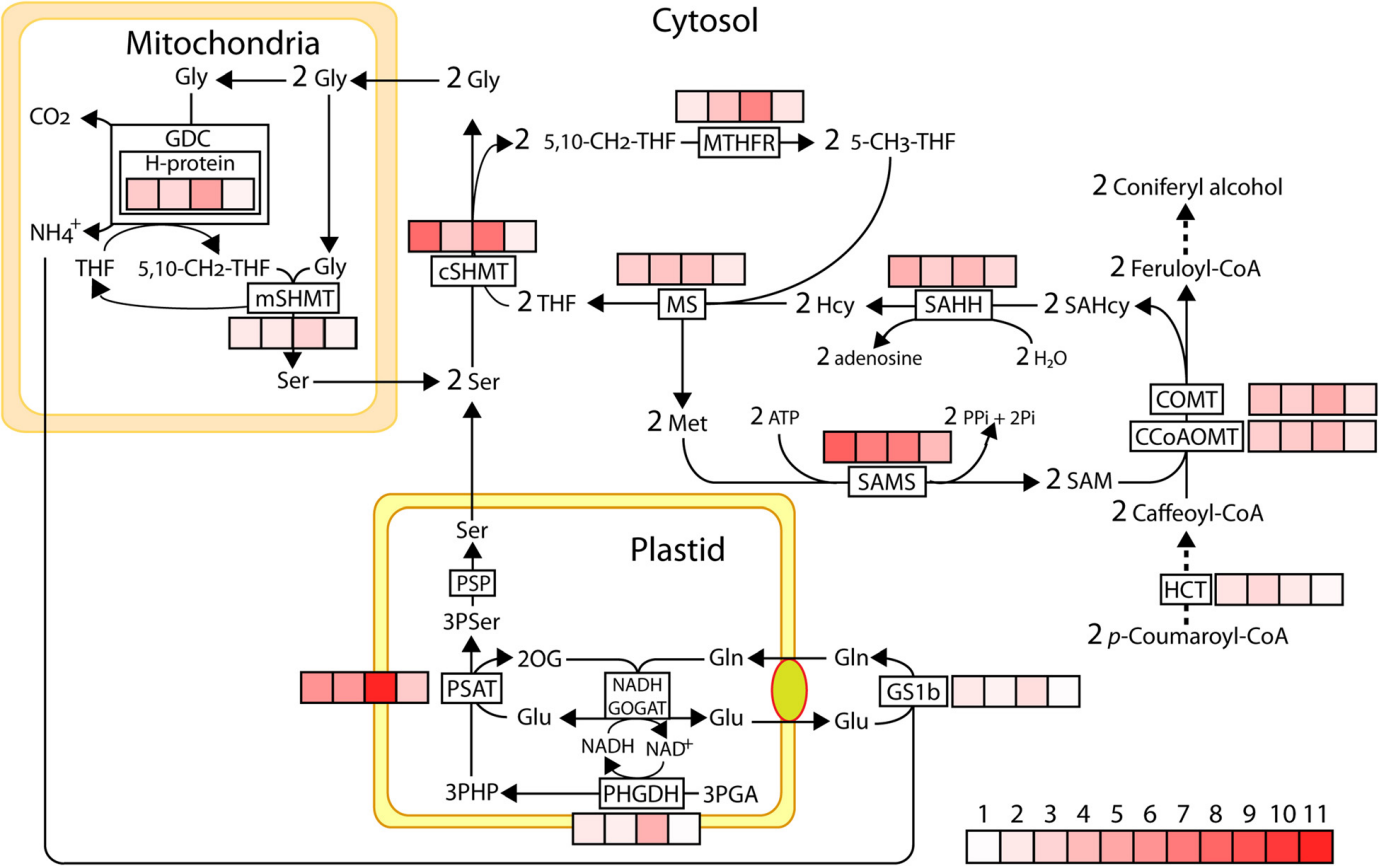
**Enzymes involved in S-adenosylmethionine metabolism and differential expression of the corresponding genes in Cx and Ox.** Transcript levels were analyzed in samples from four adult trees (T1-08, T2-08, T3-08, T4-08 in Fig. 2) after two months of artificial bending of stems, and the Cx/Ox fold change in each tree as determined by qRT-PCR is represented in four squares for every gene by a red color scale. Numerical values of fold change represented by the color scale are shown at the bottom right. GDC: glycine decarboxylase complex; mSHMT: mitochondrial serine hydroxymethyltransferase; cSHMT: cytosolic serine hydroxymethyltransferase; MS: methionine synthase; MTHFR: methylenetetrahydrofolate reductase; SAMS: S-adenosylmethionine synthase; COMT: caffeate O-methyltransferase; CCoAOMT: caffeoyl-CoA-O-methyltransferase; SAHH: S-adenosylhomocysteine hydrolase; HCT*:* hydroxycinnamoyl-CoA:shikimate hydroxycinnamoyl transferase; PHGDH: 3-phosphoglycerate dehydrogenase; PSAT: 3-phosphoserine aminotransferase; PSP: 3-phosphoserine phosphatase; NADH GOGAT: NADH-dependent glutamate synthase; GS1b: glutamine synthetase 1b; THF: tetrahydrofolate; HCy: homocysteine; SAHcy: S-adenosylhomocysteine; 3PGA: 3-phosphoglycerate; 3PHP: 3-phosphohydroxypyruvate; 3PSer: 3-phosphoserine.

To unambiguously validate the differential expression patterns observed in the microarray analyses for genes related to SAM metabolism, relative transcript levels were analyzed by qRT-PCR in Cx and Ox of four independent trees from Sierra Bermeja that were sampled in 2008 (Figure 6 shows fold change values of relative transcript abundance and values for normalized relative abundance are provided in Additional file 4). These genes encode the enzymes of the activated methyl cycle and included methionine synthase (MS: contigs 27 and 21S, singletons BX255406, BX255477, BX251773, FN257093 and FN256480), S-adenosylmethionine synthase (SAMS: singletons BX248790, FN257080, FN256463, FN256552, FN564393) and S-adenosylhomocysteine hydrolase (SAHH: contig 22S). The expression of genes encoding enzymes of the monolignol pathway branch that utilizes methyl groups provided by SAM to produce methoxylated monolignols were also analyzed, including hydroxycinnamoyl-CoA:shikimate hydroxycinnamoyl transferase (HCT), Caffeoyl-CoA-O-methyltransferase (CCoAOMT) and Caffeate O-methyltransferase (COMT). Other genes included in the analysis encode enzymes involved in the supply of methyl units into the activated methyl cycle from the amino acid serine, such as cytosolic methylenetetrahydrofolate reductase (MTHFR: singletons BX254818 and FN564392) and serine hydroxymethyltransferase (cSHMT: contig 11 and singleton FN256755), mitochondrial glycine decarboxylase complex H-protein (GDCH: singleton FN256878) and serine hydroxymethyltransferase (mSHMT: singleton BX249820), and the plastid enzymes 3-phosphoglycerate dehydrogenase (PHGDH: singletons BX251805, FN256792) and phosphoserine aminotransferase (PSAT: singleton FN257119). Finally, we also considered a gene encoding glutamine synthetase (GS1b: singleton BX253698) up-regulated in Cx according to microarray analysis (Additional file 3), which has been proposed to play a key role in re-assimilation of ammonium released from different process in developing xylem [19,35]. To unambiguously verify the identity of the encoded proteins, we cloned by RLM-RACE the full-length cDNAs for those genes that were not available in databases (see Additional file 5 for accession numbers). All these genes were indeed up-regulated in Cx compared to Ox in the four trees according to fold change values of relative transcript abundance (Figure 6 and Additional file 4). However, it is interesting to note that smaller differences in transcript levels between the two types of xylem were observed in tree T4-08. As a control, the relative transcript levels for two reference genes used in the normalization of qRT-PCR data are shown in Additional file 6.

**Figure 7:**
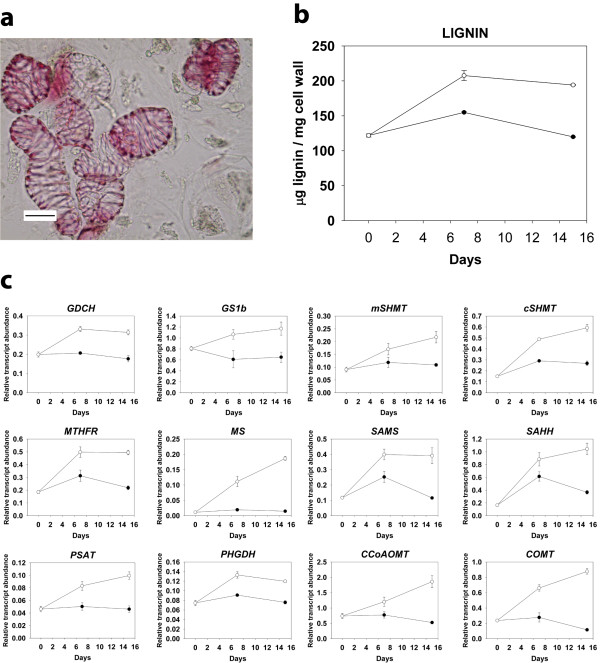
**Lignin accumulation and expression of genes involved in S-adenosylmethionine metabolism during*in vitro*tracheid differentiation from*Pinus pinaster*callus cells.** (a) Microscopic image of *Pinus pinaster* tracheids developed *in vitro*. Lignin deposited in a typical band pattern was stained with phloroglucinol. Scale bar on the bottom left represents 10 μm. (b) Lignin contents in culture of *Pinus pinaster* cells after 0, 7 and 15 days of transfer to induction medium (without hormones, open circles) or control medium (with hormones, closed circles). Values are the means of three independent quantifications ± SE. (c) Relative transcript abundance of genes involved in S-adenosylmethionine metabolism after 0, 7 and 15 days of transferring *Pinus pinaster* callus cells to induction medium (without hormones, open circles) or control medium (with hormones, closed circles). Values are the means of three independent replicates ± SE.

To further demonstrate the relationship between lignin accumulation and enhanced expression of genes related to SAM metabolism, we quantified by qRT-PCR the relative levels of transcripts in cells from *Pinus pinaster* calli that were stimulated to differentiate tracheids *in vitro*. We tested the occurrence of tracheids after 0, 7 and 15 days of transfer to the induction medium (EDM without hormones) by phloroglucinol staining and microscopy inspection (Figure 7a). Differentiated tracheids were present even in the control medium (EDM with hormones), but the number of differentiated tracheids was higher in induction medium (data not shown). Consequently lignin levels were higher in samples from induction medium (Figure 7b). Transcript levels of genes encoding enzymes involved in SAM metabolism were higher in cells transferred to induction medium compared to those transferred to control medium, in parallel with the observed increase in lignin (Figure 7c). In most of the genes the level of transcripts in cells after 15 days in induction medium was at least twice the level in cells that were cultured in control medium.

### Expression of genes encoding enzymes related to lignan-biosynthesis in cells undergoing xylogenesis

**Figure 8:**
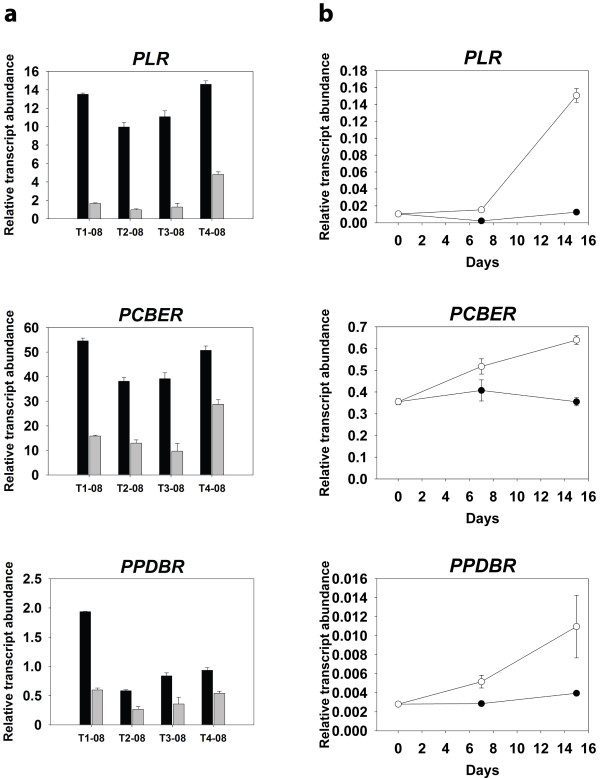
**Relative transcript abundance of three genes involved in lignan biosynthesis in cells undergoing xylogenesis.** Relative transcript abundance for each gene was determined by qRT-PCR. (a) Relative transcript levels in compression (black bars) and opposite (grey bars) developing xylem from four adult trees (T1-08, T2-08, T3-08, T4-08) after two months of artificial bending of the stems. (b) Relative transcript levels after 0, 7 and 15 days of transferring *Pinus pinaster* callus cells to induction medium (without hormones, open circles) or control medium (with hormones, closed circles). Values are means ± SE of three independent replicates. *PLR*: Pinoresinol-lariciresinol reductase; *PCBER*: phenylcoumaran benzylic ether reductase; *PPDBR*: phenylpropenal double-bond reductase.

In addition to being a lignin precursor, coniferyl alcohol is also a precursor of different lignans [36]. Three genes encoding enzymes involved in biosynthesis of coniferyl alcohol-derived lignans were also found among the up-regulated genes in Cx, according to microarray analyses (Additional file 3). Pinoresinol-lariciresinol reductase (PLR: contig 44S, singletons BX251658 and BX252917) is an enzyme involved in the 8-8’ linked lignans pathway, and phenylcoumaran benzylic ether reductase (PCBER: contigs 17 and 17S, singleton FN256729) and phenylpropenal double-bond reductase (PPDBR: contig 36S, singleton BX255256) are involved in the 8-5’ linked lignans pathway [37]. The differential expression of genes encoding these enzymes was also validated in four trees by qRT-PCR, with higher transcript levels in Cx compared to Ox (Figure 8a). Notably, this difference was also lower in tree T4-08. Transcript levels were also increased in cells of *Pinus pinaster* calli that were induced to differentiate into tracheids compared to non-induced cells (Figure 8b).

## Discussion

### Comparative transcriptome analysis shows reprogramming of cell wall related genes during compression wood formation in pine

In this work, we carried out a comparative transcriptome analysis between developing compression and opposite wood using a combination of differently constructed cDNA libraries and microarray analyses (Figure 1). Samples from three unrelated provenances, different geographic location or collected in different years (Figure 2) were integrated in the analysis to mitigate the potential effects of variability triggered by genotypic or environmental differences in order to identify a consistent set of candidate genes for wood properties.

The external stimulus that leads to the formation of compression wood caused significant changes of gene expression, as revealed by the large number of genes that were up-regulated or down-regulated in Cx compared to Ox (Figure 3) and the specific expression of some genes in Cx (Figure 4). An important component of this gene reprogramming is clearly related to the chemical and structural modifications of the thick secondary cell wall characteristic of conifer reaction wood, as suggested by the over-represented functional categories among the up-regulated genes in Cx, such as biogenesis of cytoskeleton, biogenesis of cell wall components, secondary metabolism, cellular transport and C-compound and carbohydrate metabolism (Figure 5 and Additional file 3).

A parallel up-regulation of genes encoding cellulose synthase subunits and sucrose synthase in Cx compared to Ox was observed, suggesting a higher rate of cellulose biosynthesis during compression wood formation. It has been well documented that the relative content of cellulose in compression wood is lower than in normal wood and opposite wood [21,38]. However, the S2 layer of secondary cell walls in compression wood is thicker than in normal and opposite wood, which contributes to the final thickness of the tracheid cell wall [21]. Therefore, in terms of absolute quantity, the development of compression tracheids may require higher rates of cellulose synthesis.

Different genes encoding α- and β-tubulins were also up-regulated during compression wood formation. Up-regulation of α-tubulin during compression wood formation in *Pinus taeda* has been reported previously [16], which supports our findings. Different lines of experimental evidence suggest that cortical microtubules of the cytoskeleton are involved in secondary cell wall pattern deposition and cellulose microfibril orientation [39,40]. We also identified a gene up-regulated in Cx that encodes a putative microtubule-associated protein (MAP). Several MAPs have been investigated [41] and their involvement in regulating cellulose microfibril angle (MFA) has been revealed in *Arabidopsis* MAP mutants, which show altered MFA in fiber walls [42,43]. More recently, it has been demonstrated that microtubule-associated protein AtMAP70-5 regulates secondary cell wall patterning in *Arabidopsis*[44]. Differential expression of these genes may contribute to the remarkable variation of MFA in the S2 cell wall layer between compression and opposite or normal wood, which plays a key role in the different mechanical properties of both types of wood [20,21,29,45].

Another group of genes that are up-regulated during compression wood differentiation encode enzymes that could be involved in the biosynthesis of hemicellulose, including a cellulose synthase-like, two glycosyltransferases and a xyloglucan galactosyltransferase. Although xyloglucan is a hemicellulose characteristic of primary cell walls, it has been suggested that modifications in xyloglucan may be related to the reinforcement of connections between primary and secondary wall layers [46]. In particular, the gene encoding a putative xyloglucan galactosyltransferase seems to be specifically induced during Cx formation (Figure 4b*XGT* and data not shown). In contrast, two genes encoding a putative xyloglucan endotransglucosylase/hydrolase are downregulated in Cx compared to Ox (Additional file 3, “Up-regulated in Ox” tab and Figure 4xb*XET*). Therefore, our comparative transcriptome analysis suggests specific modifications of hemicellulose biosynthesis during compression wood development.

Consistent with the enrichment of lignin in compression wood [21], we found that genes encoding enzymes of the monolignol biosynthetic pathway were up-regulated in Cx (Additional file 3, “Up-regulated in Cx” tab), such as phenylalanine ammonia-lyase (PAL), 4-coumarate:CoA ligase (4CL), p-coumarate-3-hydroxylase (C3H), cinnamyl-alcohol dehydrogenase (CAD) hydroxycinnamoyl-CoA:shikimate hydroxycinnamoyl transferase (HCT), caffeoyl-CoA-O-methyltransferase (CCoAOMT) and caffeate O-methyltransferase (COMT). In addition, the biosynthesis of monolignols demands substrates such as phenylalanine, shikimate and S-adenosylmethionine (SAM), which are provided by different metabolic reactions [12]. The quantitative importance of the shikimate and SAM metabolic pathways in the context of a highly lignified tissue, such as compression xylem, is supported by the fact that 22 of the 26 genes up-regulated in Cx that were classified in the “amino acid metabolism” functional category encode enzymes in these pathways (Additional file 3, “Up-regulated in Cx” tab).

In agreement with the elevated levels of shikimate in compression wood compared to normal wood that has been reported [30] and the higher demand of this substrate for monolignol biosynthesis, our microarray analyses showed that four genes encoding enzymes in the main trunk of the shikimate pathway were up-regulated in Cx (chorismate synthase, 3-dehydroquinate dehydratase/shikimate 5-dehydrogenase, shikimate kinase and 5-enolpyruvylshikimate 3-phosphate synthase).

### Transcriptional adaptation of SAM biosynthetic pathway to the metabolic demand of coniferyl alcohol during xylogenesis

SAM is a methyl-group donor for coniferyl alcohol biosynthesis in conifers, and for both coniferyl and sinapyl alcohol in angiosperms [34]. Genes involved in lignin biosynthesis and those encoding enzymes of the activated methyl cycle were shown to be more highly expressed in the woody core tissue of hemp [47]. According to this functional relationship, genes encoding the enzymes of the activated methyl cycle (MS, SAMS and SAHH) were also up-regulated in Cx (Additional file 3 and Figure 6). Moreover, six additional genes were also up-regulated in Cx that encode enzymes located in three different cellular compartments, which are involved in providing a continuous supply of methyl groups from serine into the cycle (Figure 6).

Two of these genes encode cytosolic serine hydroxymethyltransferase (cSHMT) and methylenetetrahydrofolate reductase (MTHFR), which catalyse the conversion of serine to glycine with the concomitant transfer of a methyl group to the tetrahydrofolate to produce 5,10 methylenetetrahydrofolate and the reduction of 5,10-methylenetetrahydrofolate (CH2-THF) to 5-methyltetrahydrofolate (CH3-THF) respectively (Figure 6). Genes encoding H-protein subunit of the mitochondrial glycine decarboxylase complex (GDCH) and the mitochondrial serine hydroxymethyltransferase (mSHMT) are also included in this functional group. The essential function of the mitochondrial glycine decarboxylase/serine hydroxyl methyltransferase complex in C1 metabolism has been previously described [48]. Glycine catabolism and recycling to serine by GDC/SHMT complex prevents the accumulation of glycine under high rates of SAM synthesis. The accumulation of glycine would push the cSHMT reaction toward the formation of serine and inhibit SAM synthesis [48]. H-protein is a critical element in the sequence of reactions in the GDC complex, acting as a mobile substrate for the other enzymatic components, and changes in the levels of H-protein may modulate the activity of the complex [49]. Different genes encoding H-protein have been found in *Populus*, and transcripts encoding individual isoforms were specifically abundant in developing xylem, suggesting the association of distinct isoforms with photorespiration and C1 metabolism [50]. Finally, genes encoding plastid enzymes 3-phosphoglycerate dehydrogenase (PGDH) and phosphoserine aminotransferase (PSAT) are involved in serine biosynthesis from 3-phophoglycerate [51], which would supply the high demand of this amino acid that is required for SAM synthesis during lignification.

The coordinated modulation of SAM and monolignol biosynthetic genes with lignin accumulation was also supported by two additional observations. First, the smallest differences in transcript abundances between Cx and Ox were observed for tree T4-08 (Figure 6), which also showed the smallest differences in lignin content (Figure 2b). Second, the relative transcript abundance of these genes during *in vitro* tracheid differentiation was parallel to lignin content (Figure 7). The increase in the levels of all enzymes involved in SAM synthesis may allow an increased flux through the pathway to supply the massive demand of methyl groups during lignification, with the minimal impact on metabolite levels [52]. In conclusion, these data strongly suggest that up-regulation of SAM metabolism genes during compression wood formation is due to an increased demand for methyl groups in coniferyl alcohol biosynthesis. However, SAM also acts as the precursor in the biosynthesis of the polyamines spermidine and spermine, the metal ion chelating compounds nicotianamine and the plant hormone ethylene. When acting as a precursor of these molecules, SAM is recycled from released 5´-methylthioadenosine through the Yang cycle [53], but genes encoding enzymes of this pathway were not found among those up-regulated in Cx.

As a result of the activity of mitochondrial GDC during lignification the release of significant amounts of ammonium should be expected (Figure 6), in addition to the ammonium released in the reaction catalysed by PAL. The massive ammonium release could compromise nitrogen economy in a woody perennial during secondary growth if it is not efficiently recycled [19]. It appears that in order to cope with the high amounts of ammonia released by PAL and GDC activities during lignification a glutamine synthetase gene (*GS1b*) was up-regulated in Cx and during *in vitro* tracheid differentiation (Figure 6 and Figure 7). This gene has been functionally associated with ammonia assimilation in vascular tissues of seedlings [35,54].

In conifers, lignin lacks sinapyl alcohol, and coniferyl alcohol is the most abundant monolignol [12] in the polymer. Compression wood is relatively enriched in H subunits, and an increase in p-glucocoumaryl alcohol is associated with development of this reaction wood [30]. Nevertheless, the increased level of transcripts for enzymes involved in SAM synthesis in Cx indicates a higher demand for coniferyl alcohol to respond to an overall increase in lignin production, which is consistent with the reported higher abundance of coniferin residues in compression wood relative to normal wood in *Pinus taeda*[30]. However, an additional fate for coniferyl alcohol is the synthesis of lignans [37]. Three genes encoding enzymes involved in coniferyl alcohol-derived lignan metabolism were also up-regulated in Cx compared with Ox and in cells that were induced to differentiate into tracheids *in vitro* compared to non-induced cells (Additional file 3, Figure 8): pinoresinol-lariciresinol reductase (PLR), phenylcoumaran benzylic ether reductase (PCBER) and phenylpropenal double-bond reductase (PPDBR). These results suggest that during compression wood formation an increased demand for coniferyl alcohol residues may also be related to an increase in the biosynthesis of lignans. Lignans have been related to defense and heartwood formation [36]. Moreover, PCBER is a prominent poplar xylem protein that is strongly associated with lignifying cells [55], and a general function for PCBER has been proposed in lignifying tissues and wood development. Lignans may be infused in the secondary cell wall during lignification to cope with the oxidative stress accompanying lignin polymerization [55], which may explain the enhanced expression of lignan biosynthesis genes during compression wood formation and *in vitro* tracheid differentiation.

## Conclusions

Our strategy for comparative transcriptomic analysis in pine Cx and Ox has revealed a broad spectrum of changes in the transcriptome of differentiating xylem cells, consistent with the formation of wood with different structures and compositions. This work provides a resource for further characterization of molecular components that determine variation in softwood properties. In particular, the coordinated modulation of expression that was observed for genes involved in serine, SAM, monolignol and lignan biosynthesis, and the central role of glutamine synthetase to avoid N deficiency are important for understanding plant metabolic requirements and regulation during intensive lignification, and should thus be considered in strategies for lignin bioengineering.

## Methods

### Plant material

To construct the different cDNA libraries for microarray manufacturing, *P. pinaster* samples of compression (Cx), opposite (Ox), early (Ex) and late (Lx) developing xylem were collected from different genotypes of a Corsican clonal population planted in 1986 in the forestry station of INRA-Pierroton (Aquitaine, France). Compression wood was induced by bending the stem at an angle of 15° away from the vertical, and 12 samples were collected at the beginning (early wood) and at the end (late wood) of the growing season in 1998 and 1999 (after 8 days, 40 days, 120 days and 1.5 year of starting the stimulus) and 2000 (after 6 hours and 1 days after starting the stimulus). Cx was sampled from the underside of the bent trunk and Ox from the upper side. Ex and Lx samples were collected from a 14-year-old straight tree in April and August 1999, respectively. Juvenile (Jx) and Mature (Mx) developing xylem were sampled in 2001 from the crown and the base (breast height) of the stem in a 30-year-old maritime pine tree from the Aquitaine provenance.

For microarray and northern hybridizations, samples of Cx and Ox were collected from four maritime pine trees between 25 and 35 years old (T1-05, T2-05, T3-05 and T4-05) in May 2005 in Sierra Bermeja (Estepona, Spain). For real-time quantitative PCR analyses (qRT-PCR), samples of Cx and Ox were collected in May 2008 in Sierra Bermeja from four different trees (T1-08, T2-08, T3-08 and T4-08). Cx was induced by bending the tree stem at an angle of 45° for 60 days before sampling. Developing xylem was scraped with a scalpel after removing bark and phloem. Samples were immediately frozen in liquid nitrogen after harvesting and stored at -80 °C until use.

### Development of *Pinus pinaster* calli and *in vitro* differentiation of tracheids

Calli were developed from *Pinus pinaster* hypocotyl explants using the procedures and culture media described by Möller *et al.*[56]. To induce tracheids, callus cells were suspended in EDM liquid medium and transferred to induction media following the same procedure as described by Möller *et al.*[56], except that the induction medium was EDM medium without hormones instead of EDM medium supplemented with activated charcoal. EDM medium with hormones was inoculated in parallel as a control medium.

### Tissue preparation and phloroglucinol staining for light microscopy

Xylem scrapings were fixed by the freeze substitution method [57]. Tissue pieces were embedded in Paraplast (Leica Microsystems) at 42 °C in a progression series with Histo-Clear and then incubated six times in Paraplast at 62 °C for 8 h. Sections (10 μm) were then obtained from embedded tissues with a Leitz microtome (Ernst Leitz, Midland, Ontario, Canada) and directly mounted onto poly-L-Lys-coated glass slides. Sections were stained with phloroglucinol (Sigma-Aldrich) following the procedure described elsewhere [58] and visualized under light microscopy, using a Nikon Eclipse E 800 microscope.

### Lignin quantification

Cell wall preparation and lignin quantification was performed following the method described by Lange *et al.*[59]. For cell wall preparation 100 mg of tissue was used.

### Isolation of RNA

Total RNA was isolated following the method of Chang *et al.*[60]. RNA concentration and purity was determined by spectrophotometry and integrity was confirmed by electrophoresis on denaturing agarose/formaldehyde gels.

### Construction of cDNA libraries

The cDNA probes for microarray construction were obtained from two different sources. A composite cDNA library was constructed from a mixed pool of equal amounts of total RNA extracted from samples of the Corsican provenance described above, including Ex, Lx and the Cx and Ox samples from artificially bent trees for different periods of time (from 6 hours to 1.5 years). PolyA(+) RNA was isolated from the mixed pool of total RNA, and the cDNA library was constructed using the λ-ZAP-cDNA synthesis kit (Stratagene, La Jolla, CA, USA).

The second source of cDNA probes was four subtractive cDNA libraries constructed from samples of the Corsican provenance by the suppression subtractive hybridization method (SSH), using the PCR-Select cDNA Subtraction Kit (Clontech, Palo Alto, CA, USA). Two SSH libraries were produced using cDNAs from Cx and Ox by performing the subtraction procedure in both directions. The same procedure was followed to obtain two SSH libraries with samples of Jx and Mx. Subtracted cDNAs were cloned in pGEM®-T Easy plasmid (Promega, Madison, WI, USA), transformed into *Escherichia coli* JM109 (Promega) or XL1Blue (Stratagene, La Jolla, CA, USA) and plated on LB plates containing 100 μg/mL ampicillin, 1 mM IPTG and 80 μg/mL X-gal. White colonies were selected from each library and separately cultured in 96-well plates containing LB with ampicillin.

### Construction of cDNA microarrays

To construct cDNA microarray 1, 2800 inserts from the composite λ-ZAP cDNA library were PCR-amplified using T3 and T7 primers or M13 Forward and M13 Reverse primers. PCR products were purified with 96-well multiscreen filter plates (Millipore Corp. Bedford, MA, USA). Amplified cDNAs were checked on agarose gel electrophoresis after purification. Solutions of purified-cDNA probes in 50% DMSO were prepared to a concentration between 100 and 200 ng/μl, and stored at -20 °C until use. Each cDNA probe was printed in duplicate onto ULTRA gaps II coated slides (Corning Inc., NY, USA) using a Qarray2 (Genetix Ltd, Queensway, UK) with a telechem printing head and 16 split pins (Biorobotics, Cambridge, UK), and with a 4 x 4 configuration. As a control, ArrayControl Sense Oligo Spots (spikes) (Ambion Inc., Austin, TX, USA) were included. After printing, the slides were dried at room temperature and the spotted cDNA were cross-linked to the slide surface by UV irradiation at 300 mJ/cm^2^.

For cDNA microarray 2, 4041 cDNA clones from the four SSH libraries described above were randomly selected and cDNAs inserts were amplified by PCR using Nested PCR primers 1 and 2R included in the PCR-select cDNA subtraction kit (Clontech, Palo Alto, CA, USA). PCR-amplified cDNA were purified and spotted in duplicate as described above.

### Microarray hybridization, scanning and data acquisition

Dye labeled aRNA was synthesized with the Amino allyl MessageAmp II aRNA amplification kit (Ambion Inc., Austin, TX, USA). Prehybridization, hybridization, and posthybridization washes were carried out using Pronto!™ Universal Hybridization Kit (Corning Inc.) in a HS 400 Pro hybridization station (Tecan Trading AG, Switzerland). Five micrograms of each of the labeled aRNA targets was added to the hybridization solution and denatured at 95 °C for 5 min. The slides were hybridized for 16 h at 42 °C. Hybridized slides were scanned with 5 μm resolution, and signal intensities were detected with a Q-Scan scanner (Genetix).

### Microarray data analysis

Four dual-target hybridizations were performed using labeled aRNA targets from Cx and Ox samples from different individual pine trees (T1-05, T2-05, T3-05 and T4-05), including two dye-swap experiments. Since every slide was printed with two full replicates of the microarray, each microarray data set consisted of four dye-balanced hybridizations for each type of xylem in duplicate. Spots flagged below 0 using GenePix v6.0 software (Axon Instruments) and those with signal intensities that did not surpass 2X the background signal in both channels were discarded. Background correction was performed with the “normexp” method of the limma library. The M value was defined as the base two logarithm of every expression ratio, computed as the ratio between the background-corrected foreground intensities of the Cy3 and Cy5 channels. Raw expression data were normalized for all sources of systematic variation with the print-tip loess [61], using the common assumption of considering the whole microarray expression as invariant. Scaling between arrays was not needed. Gene significance was then estimated using a robust linear model corrected by a moderated *t*-test (empirical Bayes) [62]. The multi-testing effect was corrected adjusting *p*-values by the Benjamini and Hochberg method [63]. A gene was considered significantly up- or down-regulated if it met these two criteria: (1) adjusted *p* ≤ 0.001; and (2) fold change ≥ 1.5. The Biobase v 2.0.1 of the Bioconductor package [64] was installed under R version 2.7.1 for all statistical analysis, mainly the limma v 2.14.5 [65] and marray v 1.18.0 libraries (http://www.bioconductor.org/packages/release/bioc/html/marray.html). The outputs of microarray analyses carried out in this study are available at Gene Expression Omnibus data repository (GEO accession numbers GSE37678 and GSE37736). All data are MIAME compliant.

### Sequencing and sequence analysis

A total of 12,134 plasmid clones from the composite cDNA library were sequenced by single pass from the 5´-end with the T3 primer, which rendered 8429 ESTs. These sequences were pre-processed and assembled into unigenes before proceeding to functional annotation as described elsewhere [32]. Sequencing of cDNA clones from the SSH libraries from genes that were differentially expressed in Cx and Ox according to microarray 2 data were carried out using M13 forward, SP6 or T7 primers. Vector and adapter sequences were deleted using SeqTrim [66]. Sequences with an internal *Rsa*I site were discarded and not considered for contig assembly, as they were supposed to contain fusions of two cDNA fragments. All the sequences were deposited in the EMBL Nucleotide Sequence Database (accession numbers are listed in Additional file 3).

Contigs were established using CAP3 [67]. Non-redundant cDNA sequences after EST assembly were annotated by BLASTX at GenBank and classified into functional groups using data reported in the literature and MIPS functional catalogue [68]. Only BLASTX hits with E-values ≤ 1x10^-10^ and a minimum of 50% similarity were selected for annotation. Sequences showing weak similarity (E-value > 1x10^-10^) and those without significant similarity were compared by BLASTN algorithm with the Pine Gene Index database (http://compbio.dfci.harvard.edu/tgi/cgi-bin/tgi/gimain.pl?gudb=pine). The resulting pine tentative contigs (TCs) or pine ESTs, which shared more than 90% of identity with each query sequence, were used as new queries for BLASTX at GenBank.

### Northern blot analysis

Seven micrograms of total RNA from Cx or Ox were analyzed by Northern blot as previously described [69]. ^32^P labeled probes of selected genes were produced with the High Prime System (Roche Diagnostics). Prehybridizations and hybridizations were performed at 65 °C. Hybridized membranes were washed with 1X SSC, 0.1% SDS and 0,1X SSC, 0.1% SDS at 65 °C.

### Real-time quantitative PCR analysis (qRT-PCR)

RNA samples were treated with RNase-Free rDNase to eliminate genomic DNA contamination and then purified using NucleoSpin RNA clean-up (Macherey-Nagel, Dürem, Germany). The quality of the treated RNA was checked by both gel electrophoresis and spectrophotometry. Reverse transcription using 1 μg of total RNA was carried out with iScript cDNA Synthesis Kit (Bio-Rad Laboratories, CA, USA).

Sense and antisense primers were designed for the specific amplification of selected genes. The sequences of the primers are shown in Additional file 5. PCR reactions were performed in a Stratagene MxPro 3000P Real-Time PCR System (Stratagene). Reactions were performed in 25 μL containing Quantimix easy master mix (Quantimix Easy SYG kit, BioTools B&M Labs, S.A., Madrid, Spain), 0.4 μM of each primer and 10 ng of cDNA (RNA equivalent). PCRs were performed by incubation 2 min at 95 °C followed by 40 cycles of 30 s at 95 °C, 30 s at specific annealing temperature and 15 s at 72 °C. Fluorescence was measured at the end of each extension step. Three technical replicates were analyzed for each sample. The specificity of each amplification reaction was verified by melting point analysis at the end of each experiment, and during protocol development by gel electrophoresis. In every assay negative controls were included with RNA not reverse transcribed from the different analyzed samples or without template to rule out contaminations due to remaining genomic DNA or sample manipulations.

The values of amplification efficiency, Ct and initial fluorescence (R_0_) for every reaction were calculated with the Miner algorithm [70]. Relative expression levels were obtained from the ratio between R_0_ of the target gene and a normalization factor. To determine the normalization factor, the relative transcript abundances of five genes with fold change value of 1 in the microarray analyses were also determined by qRT-PCR (see Additional file 5 for primer sequences) and analyzed with the geNorm algorithm [71] to select the combination of more stable genes in every experiment. For Cx versus Ox comparison the geometric mean of R_0_ obtained for ribosomal protein L34 and actin was used. For the analysis of relative transcript abundance during *in vitro* development of tracheids the geometric mean of R_0_ values for the genes encoding 40 S ribosomal protein S27, Ubi-like protein and elongation factor 1α was used.

## Competing interests

The authors declare that they have no competing interests.

## Authors' contributions

DPV, SMD-M, FMC and FRC designed and constructed the microarrays. DPV, SMD-M, E-SSS, RAC and SHEK performed the experiments. DO and FRC constructed the different cDNA libraries. RB helped in managing and arranging cDNA clones for EST generation. MGC performed the computational analyses of hybridized microarrays. DPV and FRC analyzed the data and made biological and literature interpretations of the results. FMC and MGC discussed the data and revised the manuscript. FRC designed the experiments, wrote the grants that funded the project, directed the project and wrote the manuscript. All authors read and approved the final manuscript.

## Supplementary Material

Additional file 1 **Relative expression values obtained with the analysis of microarray 1 and statistical significance.** Spots flagged as bad, not found or absent and those with a signal intensity that did not surpass 2X their background signal were discarded. The relative expression level is expressed as the binary logarithm of the Ox/Cx expression ratio (log2 Fold Change). For each probe the values for the moderated t-statistic (t), associated *p*-value (P-value) and the associated *p*-value after adjustment for multiple testing (Adjusted P-value) are shown. Click here for file

Additional file 2 Relative expression values obtained with the analysis of microarray 2 and statistical significance. Click here for file

Additional file 3 **Sequences of annotated EST and contigs of genes differentially expressed during compression and opposite wood formation.** The sequence with the higher match score in the GeneBank (GeneBank match) together with the BlastX outcome values (eValue, Score, Positives) or the Pine Gene Index database (PGI tentative contig) are included. Values of relative expression (log2 Fold Change (Ox/Cx)) according to microarray analysis and statistical significance are also shown. Click here for file

Additional file 4 **Relative transcript abundance of genes involved in S-adenosylmethionine metabolism in Cx and Ox.** Values for normalized relative abundance ± SE used to construct Figure 6. Click here for file

Additional file 5 Sequences of primers used in qRT-PCR. Click here for file

Additional file 6 **Relative transcript abundance of the reference genes encoding ribosomal protein L34 and actin in the RNA samples from Cx (black bars) and Ox (grey bars).** Values are means ± SE of three independent replicates. Click here for file
