# Supplementary material for: Reprogramming of gene expression during compression wood formation in pine: Coordinated modulation of S-adenosylmethionine, lignin and lignan related genes
Source: BMC Plant Biol. 2012 Jun 29;12:100. doi: 10.1186/1471-2229-12-100 (PMC3406974; doi:10.1186/1471-2229-12-100)
Supplement: Additional file 5 — Sequences of primers used in qRT-PCR. [file 1471-2229-12-100-S5.pdf]

Gene	Primer sequence	Orientation	GenBank Accession number of full-length cDNA sequence
GDCH	5′-ACAGATGATCTAGGACATGC-3′	Forward	
GDCH	5′-CACTTATTTGCACTTGAAGG-3′	Reverse	HE574563
GS1b	5′-TGATTGCTGAGACCAACCATCC-3′	Forward	
GS1b	5′-GGGAAATACTTTAGGCACAGAGAG-3′	Reverse	AJ005119
MS	5′-ACATGGTTGCATCTGCCAAG-3′	Forward	
MS	5′-CAAGACCACAAACCAACTAACC-3′	Reverse	HE566045
SAHH	5′-CCGTTGAGGGACCTTACAAG-3′	Forward	
SAHH	5′-TGGAACAAAACAATGGAGCCG-3′	Reverse	HE574555
SAMS	5′-CCTGGCATGATTTCTATCAACC-3′	Forward	
SAMS	5′-TGACTTGCCTCTTACTTCACAG-3′	Reverse	HE574556
mSHMT	5′-CCACACTTCCAAGCTGAAATTG-3′	Forward	
mSHMT	5′-TCAAAGCTGGTCTGTAACTCC-3′	Reverse	HE574554
cSHMT	5′-GCACAGTTTGATATGCCTGG-3′	Forward	
cSHMT	5′-ATCCCTCTAGCTACCAACTTTC-3′	Reverse	HE574564
HCT	5′-TGCAACCAGATCATATGGCTC-3′	Forward	
HCT	5′-ATCCCGAGTACAATTCTCCATG-3′	Reverse	HE574565
COMT	5′-GGCGTTAAACCTGTCTGTTG-3′	Forward	
COMT	5′-AATCGTAGTGGGGACTCTAGG-3′	Reverse	HE574557
CCoAOMT	5′-TCCCCTGAGGAAATATGTGAG-3′	Forward	
CCoAOMT	5′-CAATAGACGAGCAGAAACAAGG-3′	Reverse	FN824798
MTHFR	5′-TTGTATCCCGATGGTGATCC-3′	Forward	
MTHFR	5'-AACCACACTGTGACAGCTTC-3'	Reverse	HE574560
PHGDH	5′-AGATGACGAACCGACAAAGG-3′	Forward	
PHGDH	5′-ATGCTCTAGTTGTGCCCTGA-3′	Reverse	HE574561
PSAT	5′-TAAGGGACATCGATCAGTGG-3′	Forward	
PSAT	5′-AGCCTTGCAGATTTGAGACC-3′	Reverse	HE574562
PPDBR	5′-ACCTGTCATTTATCTGGGGC-3′	Forward	
PPDBR	5′-GGCTTGAGACAAATGACTGC-3′	Reverse	HE575885
PLR	5′-TCAATCCAACCAACTGGAGG-3′	Forward	
PLR	5′-CAAAGCAATCCTGATACGAGCG-3′	Reverse	HE574558
PCBER	5′-TACCACTGTCGATGAGTACC-3′	Forward	
PCBER	5′-ACAGGAGGTTCTGTTCTGCC-3′	Reverse	HE574559

Reference gene	Primer sequence	Orientation
L34	5'-CTAGGAGTTAGGGTTAAGATGC-3'	Forward
L34	5'-GGTACATGGTCAAAACAAGCATG-3'	Reverse
ACT	5'-ATCTCTCAGCACATTCCAACAG-3'	Forward
ACT	5'-TATTGCCACCATCATCTCAAGC-3'	Reverse
EF1 α	5'-TGCTGTTGGAGTCATCAAGG-3'	Forward
EF1 α	5'-CTCGTGCATCAGAATCAGACA-3'	Reverse
UBI-like	5'-ACGATCATGTTTTTCAACTACGC-3'	Forward
UBI-like	5'-TCATAACCATCAGAATGCCAATC-3'	Reverse
27s	5'-TCTTGAGAGTGGAGAATGGG-3'	Forward
27s	5'-CGCATCAGTCATACTCACCT-3'	Reverse
