# Supplementary figures and images for: Reprogramming of gene expression during compression wood formation in pine: Coordinated modulation of S-adenosylmethionine, lignin and lignan related genes
Source: BMC Plant Biol. 2012 Jun 29;12:100. doi: 10.1186/1471-2229-12-100 (PMC3406974; doi:10.1186/1471-2229-12-100)

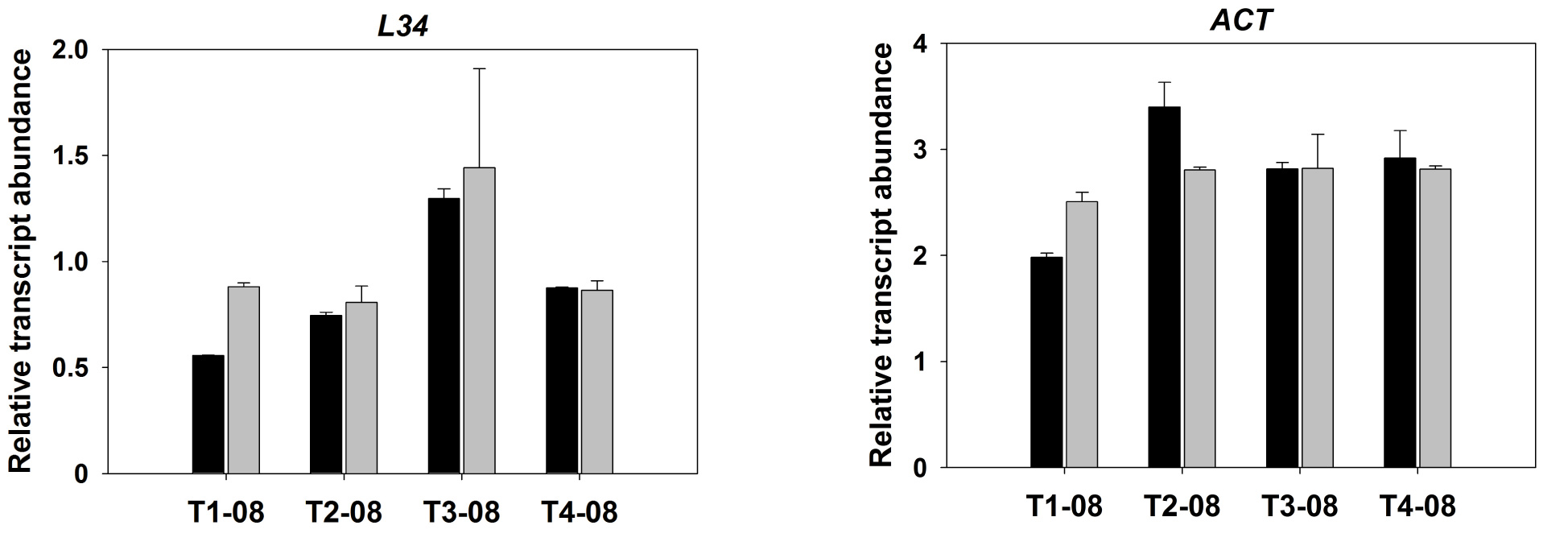

Supplement: Additional file 6 — Relative transcript abundance of the reference genes encoding ribosomal protein L34 and actin in the RNA samples from Cx (black bars) and Ox (grey bars). Values are means ± SE of three independent replicates. [file 1471-2229-12-100-S6.jpeg]
